# Using physiology to better support wild bee conservation

**DOI:** 10.1093/conphys/coac076

**Published:** 2023-01-03

**Authors:** Clementine Leroy, Jean-Luc Brunet, Mickael Henry, Cedric Alaux

**Affiliations:** INRAE, UR 406 Abeilles et Environnement, 84 914 Avignon, France; INRAE, UR 406 Abeilles et Environnement, 84 914 Avignon, France; INRAE, UR 406 Abeilles et Environnement, 84 914 Avignon, France; INRAE, UR 406 Abeilles et Environnement, 84 914 Avignon, France

## Abstract

There is accumulating evidence that wild bees are experiencing a decline in terms of species diversity, abundance or distribution, which leads to major concerns about the sustainability of both pollination services and intrinsic biodiversity. There is therefore an urgent need to better understand the drivers of their decline, as well as design conservation strategies. In this context, the current approach consists of linking observed occurrence and distribution data of species to environmental features. While useful, a highly complementary approach would be the use of new biological metrics that can link individual bee responses to environmental alteration with population-level responses, which could communicate the actual bee sensitivity to environmental changes and act as early warning signals of bee population decline or sustainability. We discuss here through several examples how the measurement of bee physiological traits or performance can play this role not only in better assessing the impact of anthropogenic pressures on bees, but also in guiding conservation practices with the help of the documentation of species’ physiological needs. Last but not least, because physiological changes generally occur well in advance of demographic changes, we argue that physiological traits can help in predicting and anticipating future population trends, which would represent a more proactive approach to conservation. In conclusion, we believe that future efforts to combine physiological, ecological and population-level knowledge will provide meaningful contributions to wild bee conservation-based research.

## Teaser

Due to growing environmental pressures on wild bee populations, there is an urgent need to better monitor population trends, identify environmental drivers of population changes and develop conservation strategies. In this paper, we discussed how physiological metrics could contribute to solving those conservation issues for wild bees.

## Introduction

Like many organisms on earth, wild bees are facing growing anthropogenic pressures, including agricultural intensification (e.g. habitat loss, lack of food resources and pesticide use), climate change and the spread of invasive species and diseases (Potts *et al.,* 2010). Consequently, in recent decades, a decline in bee species richness has been observed on all continents except Oceania (Biesmeijer, 2006; Cameron *et al.,* 2011; Ollerton *et al.,* 2014; Zattara and Aizen, 2021). In fact, according to the International Union for Conservation of Nature and Natural Resources, for which geographic range and population size are two of the criteria used for assessing conservation status, around 9% of the wild bee species in Europe could be declared as threatened, a percentage potentially underestimated given the high data deficiency for over half of the European bee species (Nieto *et al.,* 2014; Potts *et al.,* 2016). This is especially worrisome given that wild bees, through pollination, play a critical role in the maintenance and functioning of both natural and agricultural ecosystems (Klein *et al.,* 2007; Ollerton *et al.,* 2011; Garibaldi *et al.,* 2014). This crucial role in maintaining pollination services therefore provides strong arguments for promoting their conservation (Winfree *et al.,* 2018) in addition to the argument of their intrinsic value of biodiversity.

As a result, there is an urgent need to (i) better identify population trends at local or national scales, as well as their environmental drivers and (ii) develop agricultural practices that are as supportive as possible for bee populations. The evaluation of wild bee population status and trends generally relies on species records, giving an estimate of species abundance, distribution and richness (Winfree *et al.,* 2009; Zattara and Aizen, 2021). Combined with statistical modelling, such data can help in identifying drivers of bee decline. However, standardized (i.e. not opportunistic recordings by volunteers) and long-term surveys of pollinator occurrence are needed to reliably identify pollinator trends and therefore determine the impact of environmental changes (Breeze *et al.,* 2020; Garibaldi *et al.,* 2020). Interestingly, a 10-year monitoring scheme was identified as the most suitable by bee experts for detecting long-term trends in bee populations, with longer periods for assessing the influence of climate change (30 years) or landscape complexity (15 years) (Breeze *et al.,* 2020). Nevertheless, from an ecological point of view, conservation action should start before a species is endangered, i.e. before its population size declines to critical levels (Soulé, 1987). Such proactive conservation approaches, besides being more successful, are also more cost effective than reactive strategies (Drechsler *et al.,* 2011). Therefore, alternative methods that are more time-efficient and cost-effective would highly benefit the conservation of wild bee populations.

In this context, conservation biologists need biological metrics that are sensitive to environmental changes and/or suitable as early warning signals of bee population declines (i.e. before they become local or regional extinctions). For that purpose, physiology offers a promising framework to link bee responses to environmental alteration with population-level responses. First, physiology is highly sensitive and often changes quickly to allow the organism to respond to environmental disturbance. Second, physiological changes can be linked to life-history traits tightly associated with population dynamics and sustainability, such as reproductive performance and survival (Chown *et al.,* 2002; Cooke *et al.,* 2014). Accordingly, physiological changes generally occur well in advance of demographic changes and before a potential population decline (Ellis *et al.,* 2012), and therefore can help in characterizing population dynamics and its environmental constraints (Chown, 2012).

Physiological knowledge and tools, spanning a broad range of disciplines, such as endocrinology, immunology, neurophysiology, toxicology, bioenergetics and reproductive physiology, have been used for many years in solving conservation issues (Cooke *et al.,* 2013). This approach integrating physiology into the challenges of conservation has contributed to several successful stories, especially in vertebrates (Madliger *et al.,* 2016), attesting to its high potential and interest for delivering practical guidelines in conservation (Birnie-Gauvin *et al.,* 2017). Due to its potential for identifying drivers of population decline, monitoring population status and assessing the efficiency of remedial actions, we believe this approach could also benefit the field of wild bee conservation. We outline several examples of the power of physiological metrics for better understanding the influence of anthropogenic stressors and developing conservation strategies. We then highlight the key challenges that will need to be addressed for monitoring and predicting population-level changes using physiological measurements in wild bees.

### Physiology Provides a Better Understanding of the Impact and Consequences of Anthropogenic Pressures on Bees

All bees (social and solitary) have the particularity to be central-place foragers. Once adult, bees establish a nest and forage for pollen and nectar around that nest for their entire life. Contrary to animals that move from place to place for collecting resources, the foraging distance of bees is limited to a specific range around the nest, which is generally related to their body size (Greenleaf *et al.,* 2007). This has strong implications for their populations, since bee survival and fitness will be determined by the habitat quality in a specific range around their nest (e.g. floral resource availability, pollution, etc.), which makes them particularly sensitive to changes in these habitats. It is therefore crucial to better understand how anthropogenic pressures leading to changes in habitat quality affect bees, before developing management strategies to minimize or reverse current population trends of species of concern. In this regard, physiological markers measured in response to environmental stressors or habitat quality can reveal mechanisms that contribute to population decline and thereby provide a cause-and-effect relationship between environmental characteristics and bee survival (Tracy *et al.,* 2006). Here we offer examples of the importance of physiology for better evaluating the risks posed by anthropogenic pressures in bees. We focus on the main threats that may act at large scales and affect most of bee taxa, such as environmental chemicals, decline of floral resources and climate change.

### Environmental chemicals

Landscapes have been largely transformed by agronomic practices and one of the most striking consequences is the widespread use of agrochemicals, which have permeated the biosphere causing exposure to many non-target organisms. To investigate the potential effects of such exposure on non-target organisms, ecotoxicology has emerged as a leading discipline and is perhaps the oldest discipline linking physiology to questions of conservation (Stevenson *et al.,* 2005). The most popular and precursory example is undoubtedly the discovery of the inhibition, by the chemical dichlorodiphenyltrichoroethane, of calcium carbonate deposition in eggshells of birds of prey, leading to their reproductive failure (Grier, 1982). Bees are also exposed to pesticides as multiple residues can be found in pollen and nectar of crops and wild flowers (Botías *et al.,* 2015; Rundlöf *et al.,* 2015). To analyse the consequences of bee exposure to pesticides, ecotoxicological studies have traditionally assessed the lethal effects. However, more recently, a large body of evidence has shown that this approach underestimates the risks related to low environmental doses and current research methods generally integrate sublethal effects into toxicological studies (Sandrock *et al.,* 2014; Sgolastra *et al.,* 2018; Brandt *et al.,* 2020; Lehmann and Camp, 2021; Strobl *et al.,* 2021).

Sublethal exposure to environmental chemicals can be investigated by the use of biomarkers of effects, which are defined as any measurable biochemical, physiological or genetic variation that provides evidence of exposure to and/or effects of one or more environmental pollutants (Mouneyrac and Amiard-Triquet, 2013). Such biomarkers enable one to determine whether and what type of exposure took place and have proven to be promising tools for evaluating ecotoxicological health status and monitoring environmental quality of honeybees (*Apis mellifera*) (Badiou-Bénéteau *et al.,* 2012; Caliani *et al.,* 2021). Applying this biomarker approach to *Osmia* and *Bombus*, which have been recently proposed as additional test surrogates to honeybees (European Food Safety Authority, 2013), but also to other bee genera, would greatly improve the evaluation of past and present exposure to anthropic contamination and determine habitat quality for wild bee populations. This was confirmed by a study, which found an increase in acetylcholinesterase expression in bumblebees (*Bombus impatiens*) foraging in agricultural areas using neonicotinoid crop protection (Samson-Robert *et al.,* 2015). More recently, another study identified genes differentially expressed between *Bombus terricola* worker bees sampled in agricultural and non-agricultural sites (Tsvetkov *et al.,* 2021). These differentially expressed genes significantly overlap with those found in honeybees exposed to insecticides, suggesting an exposure of *Bombus terricola* to insecticides in the studied sites. Changes in the expression of several genes upon exposure to neonicotinoids have also been identified in *Osmia bicornis* (Beadle *et al.,* 2019), which could potentially provide biomarkers of field exposure to pesticides for this solitary bee species. An alternative and more specific approach is the multi-biomarker approach that is widely used in various aquatic and terrestrial organisms to detect and evaluate the effects of exposure to chemical contaminants. By testing and analyzing variations of several biomarkers (often enzymatic activities) upon exposure to a chemical in the laboratory or in the field, it is then possible to calculate a simple ‘Integrated Biological Response’ that is specific to the chemical and its concentration (Beliaeff and Burgeot, 2002). This has notably been developed successfully in honeybees for discriminating exposure to different concentrations of Cadmium and the fungicide Amistar® Xtra (Caliani *et al.,* 2021).

Another key aspect of pesticide risk assessment is to determine whether such exposure leads to irreversible modifications of bee physiology that might translate into damage to bee populations. A consistent decline in the production of new queens by colonies of *Bombus terrestris* has been reported upon exposure to neonicotinoids (Whitehorn *et al.,* 2012). Whether this lowered reproduction is due to reduced pollen foraging (Feltham *et al.,* 2014), to direct pesticide effects on queen physiological development or to both is not known, but, in any case, it could have severe consequences for bumblebee populations. Physiological impairments, such as immunosuppression and reduced thoracic temperatures in *O. bicornis* and alteration of mitochondrial functions in *B. terrestris*, have also been identified in response to pesticide exposure (Azpiazu *et al.,* 2019; Colgan *et al.,* 2019; Brandt *et al.,* 2020). However, perhaps the most striking effects are the decrease in ovary development, male fertility and total offspring production, as well as a male-biassed sex ratio and higher mortality of eggs and larvae (Sandrock *et al.,* 2014; Sgolastra *et al.,* 2018; Franke *et al.,* 2020; Minnameyer *et al.,* 2021; Strobl *et al.,* 2021). These latter effects clearly suggest mechanisms that explain the observed decline of solitary bee populations in conventional agroecosystems (using pesticides) (Rundlöf *et al.,* 2015). Finally, it was recently found in bumblebees and fig wasps that high ozone concentrations can decrease the antennal sensitivity, and consequently, the attraction to floral volatile organic compounds (Vanderplanck *et al.,* 2021a). This physiological impairment might directly affect plant–pollinator chemical communication and, consequently, bee survival. All of these examples clearly illustrate the interest of studying physiologically related endpoints for the risk assessment of environmental chemicals in wild bees.

### Landscape resources

All bees have in common a reliance on floral pollen and nectar for their growth and survival. Nectar contains carbohydrates fuelling their energetic demands, and pollen provides all of the proteins, amino acids, lipids, sterols and vitamins required for their ovary development and larval diets (Roulston and Cane, 2000; Leach and Drummond, 2018). However, a decrease in the diversity and abundance of floral resources in agricultural landscapes may generate a nutritional stress for bees (Goulson *et al.,* 2015; Vaudo *et al.,* 2015), especially for wild bees, who are generally more selective for pollen nutrition than honeybees, who collect pollen from a wide spectrum of plant species (polylecty) (Galimberti *et al.,* 2014; Danner *et al.,* 2017). For instance, bumblebees seem to favour pollen quality over quantity (Leonhardt and Blüthgen, 2012), while some solitary bees have a rather specialized pollen diet by collecting pollen from a limited number of plant species (oligolecty) (Müller, 1996; Cane and Sipes, 2007; Müller and Kuhlmann, 2008).

By investigating the influence of pollen diet abundance, quality and diversity on bee physiology, several experimental studies have attempted to identify what might represent a nutritional stress for bees. It is now well established that the protein and lipid contents of pollens have a significant impact on bumblebee immunity, development and survival (Vanderplanck *et al.,* 2014; Roger *et al.,* 2017). If pollen diversity might not provide an added value *per se* (as compared to higher quality monofloral pollens), it might help to mitigate the negative effect of unfavourable pollens (Moerman *et al.,* 2017; Carnell *et al.,* 2020). The benefit of pollen mixing was notably demonstrated by Eckhardt et al. in the pollen-generalist solitary bee *Osmia cornuta* (Eckhardt *et al.,* 2014). Pure *Ranunculus* pollen had a negative effect on larval development and survival and adult body mass of both males and females, but when not predominant (less than 50% of the pollen diet), bees remained unaffected. In addition, decreased access to resources has been shown to reduce reproductive performance of solitary bees and bumblebees (Peterson and Roitberg, 2006; Cane, 2016; Centrella *et al.,* 2020; Requier *et al.,* 2020). Such data might explain the lower bee abundance and diversity observed in agricultural systems embedded in simple landscapes or with low levels of semi-natural habitats (Kennedy *et al.,* 2013; Lichtenberg *et al.,* 2017).

Finally, bee body traits (e.g. body mass and size) and nutritional health indicators (e.g. lipid content) may be useful for predicting the vulnerability of species, or at least an actual response, to changes in landscape-level floral resources. For instance, by measuring the mass, size and lipid content of several bumblebee and sweat bee species sampled in the field, Smith et al. (2016) and Stein et al. (2020) could identify which species were affected by which grassland management practices in agricultural landscapes. Body size, which is strongly affected by pollen nutrition during larval development, has also been used as a proxy for assessing the response or sensitivity of wild bees to resource availability along landscape gradients. Results vary across bee species, diet breadth and landscape types, making it difficult to draw general conclusions on a local scale (Renauld *et al.,* 2016; Banaszak-Cibicka *et al.,* 2018; Reeth *et al.,* 2018). However, two meta-analyses, performed on museum samples from the northeastern United States and the Netherlands, found that a large body size is among the traits that are negatively related to population trends (Bartomeus *et al.,* 2013; Scheper *et al.,* 2014), which is probably due to the greater pollen needs of larger bee species combined with food limitation. Altogether, these experimental and survey data highlight nutritional stress as a driver of bee decline and identify, for some species, which specific diets might be unfavourable.

### Climate change

Since bees, as insects, have limited ability to regulate their body temperature, their physiology, behaviour and survival are largely constrained by environmental temperatures. Determining and predicting the consequences of climate change on bees therefore has become a growing research topic. To quantify the sensitivity of bees to temperature, several studies have measured the consequences of artificial or environmental variations in temperature on physiological traits. For instance, changes in the queen body size of different bumblebee species have been linked to an increase in the mean annual minimum temperature (Gérard *et al.,* 2020). However, a large body of literature has focused on the effect of temperature on completion of the winter diapause, likely because insects generally rely on low temperatures to reduce their metabolism and preserve their energy storage until the next spring (Brown *et al.,* 2004; Williams *et al.,* 2015). Climate change is therefore expected to increase energy expenditure during the diapause, which can lead to vulnerable bees in the spring. Although bee species differ in their responses (Fründ *et al.,* 2013), this hypothesis has been verified many times, with natural or artificial pre-winter and winter conditions causing a shorter diapause duration and a decrease in fat body, body weight and consequently winter survival (Bosch and Kemp, 2004; Sgolastra *et al.,* 2011; CaraDonna *et al.,* 2018). Characterizing the baseline of overwintering gene expression, as it was done for the small carpenter bee *Ceratina calcarata*, could also help to better understand adaptations to overwintering and molecular responses to climate change (Durant *et al.,* 2016).

Variations in summer temperatures have been investigated as well and have been shown to affect voltinism in a solitary bee (*Osmia iridis*) (Forrest *et al.,* 2019). Under controlled conditions, increased temperatures were found to decrease prepupal weight and the duration of all development phases in *O. bicornis* (Radmacher and Strohm, 2011), and reduce wing size in *B. terrestris* (Gerard *et al.,* 2018). Heat shock stress also has a negative impact on bumblebee reproduction by impairing sperm viability, sperm DNA integrity and the production of pheromones involved in male attractiveness, but interestingly, this was observed only in the tested declining species (not in the widespread and warm-adapted species) (Martinet *et al.,* 2021b). Similarly, a study showed that two invasive bee species in Fiji tend to be more resistant to thermal and desiccation stress than an endemic species, which might contribute to a higher resilience to climate change (da Silva *et al.,* 2021). Warming summers can also lead to changes in bee morphology as evidenced by the decrease in tongue length of two alpine bumblebee species within 40 years (Miller-Struttmann *et al.,* 2015). Interestingly, a decrease in the abundance of long-tongued bumblebees and an immigration of short-tongued species from lower altitudes have been reported during the same period (Miller-Struttmann *et al.,* 2015). While the underlying mechanisms are not clear, it shows some phenotypic plasticity in response to climate (De Keyzer *et al.,* 2016). Last but not least, data on the thermal physiology can be extremely useful for understanding and/or predicting how different species might respond to climate stress. This was the case with the identification of bumblebee critical thermal limits, which correspond to the temperatures at which an organism may encounter failure or loss of essential functions (e.g. motor and respiratory). Measurements of thermal tolerance helped to explain population declines and range shifts in relation to climate change (Oyen *et al.,* 2016; Zambra *et al.,* 2020; Martinet *et al.,* 2021b), as well as local adaptation in bumblebees (Pimsler *et al.,* 2020). In sum, documenting variations of physiological and body traits in response to temperature changes proves to be extremely valuable for better understanding the temporal and spatial shifts in bee distribution.

### Physiology Can Help in Designing Conservation Practices

To mitigate or combat habitat loss and therefore the decline of wild bee populations, conservation efforts have been focusing on the provision of consistent foraging and nesting resources in human-dominated areas. Large-scale actions have been developed for that purpose. In Europe, the European Agri-Environmental Schemes promote environmentally friendly practices in farmlands (e.g. implementation of flower strips and hedgerows in field margins, restoration of pastures and meadows or organic farming) (Scheper *et al.,* 2013). In the United States, the Conservation Reserve Program supports the conversion of croplands into long-term conservation habitats with a recent priority given to the promotion of forage for pollinators (Osteen *et al.,* 2012).

To further improve such conservation practices, high-resolution data are needed on the nutritional needs of bee species. Studies first attempted to identify the minimum ‘pollen budget’ for maintaining a self-reproducing population (Müller *et al.,* 2006; Larsson and Franzén, 2007). However, most of the research efforts are now focusing on the determination of foraging preferences and diets that are most appropriate for bees. Among others, preferred pollen protein:lipid macronutrient ratios have been identified in *B. impatiens* and linked to bumblebee colony health and fitness (Vaudo *et al.,* 2016). Some of the minimum nutrient requirements for bee development have been described as well for several species (Vanderplanck *et al.,* 2014; Moerman *et al.,* 2016; Barraud *et al.,* 2022) and nutritional physiology has shed light on stage-specific nutritional limitations in the bee life cycle (Woodard *et al.,* 2019). Physiological markers can also provide helpful information on the interface between bee populations and their resources. For instance, by characterizing the elemental composition of bees (female, male, adult, larva) and their resources, ecological stoichiometry allows one to determine whether there is a mismatch between the elemental demands of bees and floral resources, potentially leading to an imbalanced diet (Filipiak, 2018). By using this approach, Filipiak et al. reported some stoichiometric mismatches between larvae of *O. bicornis* and particular pollen species and identified specific elements related to bee fitness (Filipiak, 2019; Filipiak and Filipiak, 2020; Filipiak *et al.,* 2021). Assessing bee nutritional requirements is therefore also a promising field of research for tailoring conservation measures to bee communities. In summary, by studying the bee–nutrient floral networks, we can identify the preferred floral species as well as the nutrients or nutrient ratios important for specific bees and the plant species providing them. Such information can then be used to adjust floral enhancement schemes or support the conservation of key plant species for bees (i.e. preferred floral species or with high nutritive values for bees) (Woodard, 2017; Parreño *et al.,* 2022).

Besides the identification of nutritional needs, assessing the efficiency of habitats designed for promoting the sustainability of bee populations is also essential. Most of the research has focused on the attractiveness of these habitats by quantifying the potential positive effects on bee abundance and species richness (Scheper *et al.,* 2013). However, the creation of floral hot spots highly attractive to a broad range of bee species might have negative side effects on bee health. Indeed, in landscapes with low amounts of semi-natural habitats, wildflower fields seeded to boost bee populations in field margins might create hot spots for parasite transmission (Piot *et al.,* 2019). In addition, such wildflowers can get contaminated with pesticides (under non-organic farming practices) and thus prolong exposure to pesticides beyond the treated crop flowering (Botías *et al.,* 2015). Therefore, in addition to attractiveness data, physiological metrics of bee health may be used as complementary criteria to evaluate the efficiency of habitats designed to be pollinator-friendly and develop Habitat Suitability Indices (HSI) to be used as operational decision support by conservationists and land-managers. HSIs are widely used tools in applied conservation (Larson *et al.,* 2004). However, physiological factors are seldom implemented into HSI algorithms, which yet is critical regarding physiological constraints faced by central place foragers (Chudzinska *et al.,* 2021). As a prerequisite, one should objectivate the links between environment and physiology in the taxonomic group of interest. In this context, physiological measures have been successfully used in honeybees to demonstrate the beneficial effects of floral landscape enrichments and uncultivated forage (e.g. pasture, grasslands, woody areas and hedgerows) on bee health (Smart *et al.,* 2016; Alaux *et al.,* 2017; Dolezal *et al.,* 2019). A few studies have shown that several markers of nutritional state in bumblebees could be promising for monitoring bee health and assessing habitat quality (Smith *et al.,* 2016; Wang *et al.,* 2019). However, further field studies with wild bees are required to provide a proof-of-concept of this ecophysiological approach for evaluating how well remedial actions are working.

### Physiology for Predicting Population-Level Changes

The measure of physiological traits is highly valuable in the identification of the sources and consequences of stressors on bees, which inform conservation actions. However, field monitoring, which consists of tracking the population status of species of concern, also plays a key role in conservation strategies (Nichols and Williams, 2006). The quantitative information that originates from monitoring programs can guide decision-making for environmental management and rehabilitation, and then help to evaluate the success of management actions. The only concern is that conventional monitoring (e.g. estimating population size) ‘is by its very nature post hoc—that is, it can only tell us what has already happened’ (Keddy, 1991). Predicting and anticipating future population changes or sustainability would represent a more powerful approach to conservation. In this context, early indicators of individual fitness, such as physiological traits, have the potential to predict organismal responses to environmental changes, and therefore anticipate changes or stability in wild bee populations. However, several challenges first need to be addressed for incorporating physiological biomarkers into conservation monitoring actions.

### Linking variations in physiology to changes in population dynamics

Biomarkers or signatures of stress might not always be indicative of declining populations, in particular when physiological changes have little effects on the organism (e.g. sublethal effects), or compensatory mechanisms allow the organism to ultimately survive and reproduce or tolerate changing environments (Van Dievel *et al.,* 2016). Furthermore, upon exposure to high levels of stress, the population may be able to persist by relying on the most physically fit or healthiest individuals (Fefferman and Romero, 2013). Therefore, to determine whether a physiological biomarker can be used as a monitoring tool for the management of wild bee populations, the most suitable approach would be to confirm that such a biomarker can be linked to proxies of individual fitness or demographic changes (Bergman *et al.,* 2019).

First, studies that simultaneously connect environmental, physiological and population metrics should be carried out. Several biomarkers, such as bee body conditions based on morphometric variables (e.g. weight, body size, body parts size), could be used since they are affected by both genetic and environmental factors that have operated through physiological mechanisms during larval development (Nijhout, 2003). These body variables are easy to acquire and potentially non-lethal and have been found to be related to population trends (Bartomeus *et al.,* 2013; Scheper *et al.,* 2014). However, in several cases they might be less informative about the type of stressor (Gerard *et al.,* 2018). Alternatively, the assessment of biochemical and genomic markers require sacrificing several specimens but are expected to provide more specific information on the stressor and its severity (Badiou-Bénéteau *et al.,* 2013; Woodard *et al.,* 2015; Kent *et al.,* 2018; Wang *et al.,* 2019). However, the lack of multidisciplinary expertise in longitudinal surveys of wild bee populations might have prevented so far the realization of studies linking eco-physiological data with population-level metrics for wild bees, as seen for honeybees (Smart *et al.,* 2016; Alaux *et al.,* 2017). Nonetheless, this could be overcome by sampling bees in monitoring surveys and integrating candidate biomarkers (e.g. bee size, weight or nutritional health indicators) into datasets of bee population demography (e.g. abundance, growth rate). Identifying individual physiological biomarkers that correlate to population trends may then help to evaluate species resilience and responses to environmental changes.

Secondly, studies could also determine whether physiological biomarkers that were already found to be affected by environmental variables are also related to some component of individual fitness (survival, reproductive performances). For instance, body size in bees, which is highly correlated to the amount of food ingested (during larval stage) and therefore to the abundance of floral resources, has been found to be positively related to female fecundity in different bee species (Bosch and Vicens, 2006; O’Neill *et al.,* 2014; Slominski and Burkle, 2021). These relationships highlight body size as a promising biomarker for monitoring population trends. However, further research is needed given that the relationship between body size and female fecundity is not consistent across studies and may depend on species and/or local environmental contexts (Bosch and Vicens, 2006).

Thirdly, comparing baseline levels of physiological biomarkers in both a decreasing and an increasing or stable population could help in determining whether a biomarker could be used to measure demographic changes (Bergman *et al.,* 2019). For instance, data about heat stress sensitivity have been especially convincing for explaining population trends and restricted habitat types in bumblebees. By measuring the physiological thermal limit of individual bees, a direct link between the thermotolerance of bumblebee species and their distribution area was found (Martinet *et al.,* 2015, 2021a; Oyen *et al.,* 2016) and widespread species proved to be less sensitive to heat stress than declining species (Zambra *et al.,* 2020; Martinet *et al.,* 2021b). Similarly, physiological thermal limits were found to be a strong predictor of bee population response to heat in urban areas, with those bees having the lowest critical thermal maximum showing the most population decline with warming (Hamblin *et al.,* 2017). Altogether, these studies showed that species’ physiology could shape community composition, and therefore help in predicting their future trends under changing environmental conditions.

### Including physiological performances into ecological niche modelling

Ecological niche modelling has become an important tool for inferring species distribution and habitat suitability. By linking the observed distribution of a species to environmental data, the correlative ecological niche models have the advantage of requiring little information on the mechanistic links between the organism and its environment and are therefore especially useful for species that are poorly studied. For instance, ecological niche modelling allowed for the prediction of overall range losses under climate change for 30 North American bumblebee species, even for species with high dispersal abilities (Sirois-Delisle and Kerr, 2018). However, such models are constrained by the availability of detailed data on species distribution, and when extrapolating species distribution to future environmental conditions (not yet experienced by species), mechanistic ecological niche models that are parametrized with physiological data are expected to be more robust (Kearney and Porter, 2009; Evans *et al.,* 2015). Notably, the inclusion of information on physiological limits of major life history characteristics (e.g. survival, growth and reproduction) can help to make inferences on species’ range limits and to understand the environmental characteristics defining the species range. This mechanistic modelling was elegantly used as a tool for the ecosystem restoration of four insect pollinators at a local scale in Australia (Tomlinson *et al.,* 2018). By projecting data of metabolic rates and thermotolerance of these pollinator species onto high-resolution topoclimatic models, the authors showed the importance of mechanistic models fitted with physiological data in providing evidence-based guidance for ecological management programs. Finally, individual- or agent-based models, by simulating the fate of individuals with respect to their behavioural and/or physiological traits, might represent an alternative to better predict population and community changes. They can incorporate any number of individual-level mechanisms and take into account their changes through time (Grimm and Railsback, 2005; DeAngelis and Grimm, 2014). Agent-based models have notably been used for exploring potential benefits of bee-friendly farming measures on the population dynamics of six bumblebee species (Becher *et al.,* 2018). However, using such these modelling approaches to predict the resilience and trends of bee populations in changing habitats implies that physiological limits of species of concern are readily available. Therefore, these knowledge gaps need to be addressed in the future to strengthen the implementation of mechanistic models. For instance, data on thermal limits and energy balance would be particularly helpful to better anticipate the persistence of population under climate and resource changes.

### Surrogate species for community-based monitoring

With over 20 000 known bee species in the world, it appears virtually impossible to develop a species-by-species monitoring approach for the prediction of population trends. This is also the case for the monitoring of bee communities in specific habitats, in particular within the goal here to use early indicators (physiological markers) of population dynamics. Measuring physiological traits of all species within a community can quickly become expensive and time consuming, and it might not always be possible to sample all species or a sufficient number of specimens per species for assessing population trends. In addition, the ecology and biology of most of wild bee species are still poorly understood. Therefore, one solution to fill these gaps would be to focus on some species that can be easily monitored and for which we could standardize relevant physiological traits measurement in order to inform on the community’s status and thus apply them in conservation contexts.

For that purpose, conservation biologists have showed a growing interest in the identification of surrogate species indicative of the state of species communities or ecosystems (Fleishman *et al.,* 2000, 2005). Surrogate species notably include indicator and umbrella species (Caro and Girling, 2010); the latter focusing on species that have large area requirements and for which conservation offers protection to species that co-occur in the same habitat (Wilcox, 1984). However, empirical tests of umbrella species validity have provided equivocal results, in part due to the focus on a large home range, which might hinder their applicability (Ozaki *et al.,* 2006; Branton and Richardson, 2011). Consequently, the concept of local umbrella species, that occupy a more spatially limited area, has been introduced by (Caro and Girling, 2010). In addition, the efficacy of putative umbrella species may depend on the co-occurring taxonomic diversity that is targeted, since umbrella species are likely more indicative of elevated biodiversity within their own taxonomic groups than across taxa (Sattler *et al.,* 2014). Because here, the goal is to monitor and predict community changes of wild bees that inhabit relatively limited areas, efforts should rather be made on the identification of local umbrella species specifically indicative of their own community. A first attempt has been made in urban areas of the Central Swiss Plateau and lowland areas of southern Switzerland by analyzing 139 bee species (Sattler *et al.,* 2014). By using several functional traits, such as dietary specialization (oligolectic and polylectic) and nesting habits (miner-carder, renter, carpenter, mason), they were able to identify six6 umbrella species (*Andrena bicolor*, *Heriades truncorum*, *Hylaeus nigritus*, *Hylaeus sinuatus*, *Lasioglossum calceatum*, *Psithyrus barbutellus*), which were indicative of bee functional biodiversity (i.e.i.e., dietary specialization, nesting habits).

An alternative or complementary approach is to target a species or a group of species whose status (abundance, population dynamics) rather reflects the environmental conditions (Carignan and Villard, 2002). The selection of such species, named indicator species, is more focused on their ecology and life history traits, and as such, they can be used as a proxy to determine the health of an ecosystem and provide early warning signals about environmental changes. Interestingly, within the aim of better supporting conservation strategies for bumblebee species in China, a recent study identified, among the 125 studied species, 26 species as indicator species of the different biogeographic regions: 14, 13, 12 and 12 species were associated with the regions of South China, North China, the Mongolian Plateau and the Tibetan Plateau, respectively (Naeem *et al.,* 2020). Although no empirical test was performed, these first studies suggest that some bee species could potentially be assimilated as surrogate species to monitor bee communities and/or environmental conditions in their respective biogeographic ranges.

Detailed studies of physiological health indicators on surrogate species (see Linking variations in physiology to changes in population dynamics) should then provide valuable information on the conditions of the environment where they are found, and consequently, for other co-occurring bee species or larger bee communities. Notably, building standard biomarkers values for these reference populations should help identifying poor or declining environmental conditions (when biomarkers deviate from the baseline or average values), especially if these biomarkers can be linked to proxies of individual fitness or demographic changes. Such species could therefore facilitate monitoring programs (a single or a few species to monitor), especially within the objective to perform proactive conservation with early indicators of species community changes.

**Figure 1:**
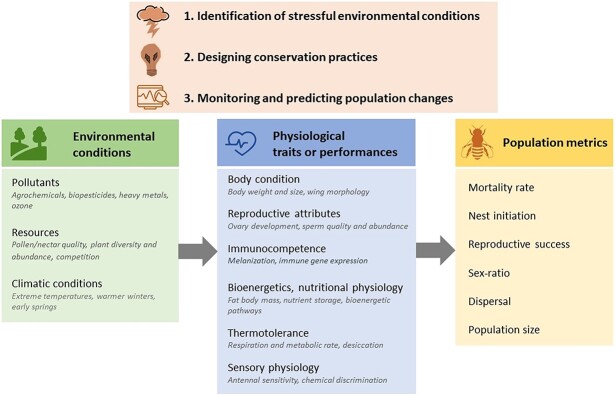
Physiological metrics in the conservation of wild bees. By linking environmental conditions to population responses, the measurement of bee physiological traits or performances can help in identifying stressful environmental conditions, and therefore in predicting population trends. Information on physiological needs can also guide conservation strategies. Main and sublists are inexhaustive examples of parameters that have been investigated in conservation-based research of wild bees.

### Dealing with time effects

In some cases, adverse environmental conditions can affect the survival or fitness of the studied organism in a subsequent season (Harrison *et al.,* 2011). If not taken into account, these carry-over effects can lead to erroneous conclusions about the effectiveness of conservation efforts (O’Connor and Cooke, 2015). Such carry-over effects have been described in honeybees, with an increase of winter mortality risks in colonies that have experienced a pollen shortage the previous season (Requier *et al.,* 2017). But they have also been documented in wild bees. By exposing, adult bees of *Osmia lignaria* to the neonicotinoid insecticide imidacloprid, researchers demonstrated a reduction of the offspring fitness (reproduction) the following season due to maternal and/or larval effects (Stuligross and Williams, 2021). Carry-over effects can also be observed at the level of bee communities’ since their abundance is typically influenced by the floral resource availability of the previous year’s (Potts *et al.,* 2003; Le Féon *et al.,* 2013). This phenomenon is due to the specific life cycle of most wild bees: females generally emerge in the spring, mate, and then provision their nests with pollen to feed their larvae, which develop into adults in the autumn and emerge the next spring. As a result, the number of offsprings, as well as some of their morphological and physiological traits, may be affected by the environmental conditions of the previous year. For instance, their body size should reflect the amount and quality of pollen resources ingested as a larva the previous spring/summer (Chole *et al.,* 2019). Similarly, diet quality may affect resource assimilation and allocation during larval development and consecutively some of the physiological processes essential to bee fitness (Filipiak *et al.,* 2021). The specificity of bee species life cycle needs therefore to be taken into account to deal with such carry-over effects and better interpret the influence of environmental factors on bee health.

Finally, physiological traits can vary in their response time to environmental changes, with some markers being more sensitive or responsive over shorter timescales than others (within minutes) (Bergman *et al.,* 2019). But also, the level of some physiological traits can naturally vary over time during the organism development or maturation. For instance, ovary development and the level of vitellogenin, the precursor of major yolk proteins in insects, gradually increases after the emergence in the mason bee *Osmia cornifrons* to reach a peak at 6 days and then decline (Lee *et al.,* 2015)*.* Physiological markers could therefore induce some biases in the way they reflect environmental conditions if time changes are not taken into account. For such typical traits, a specific and consistent monitoring should be performed, rather than a single or random sampling over time, so as to acquire thorough time series databases.

## Conclusions

Understanding and undertaking a comprehensive mitigation of the impact of anthropogenic pressures on wild bee populations is currently a demanding research challenge due to the large gaps in evaluating bee sensitivity to environmental changes (López-Uribe *et al.,* 2020), as well as in monitoring the status and trends of populations (Lebuhn *et al.,* 2013). However, we showed here, through several examples, that physiological metrics can help solve several issues related to the conservation of wild bees (Fig. 1). Indeed, the measure of physiological traits or performances, by providing a cause-and-effect relationship between environmental characteristics and bee survival, can help in identifying stressful environmental conditions. The documentation of species’ physiological needs and limits can also offer valuable information for identifying and testing the efficiency of remedial actions. Equally important are physiological metrics that can be used as early indicators of population decline. Incorporating such physiological metrics into monitoring programs is still uncommon but promising for developing a more proactive approach to conservation (Madliger *et al.,* 2018). Future efforts should however be made in the identification of physiological metrics for assessing bee population status or dynamics. To reach this goal, the identification of generalized stress biomarkers in insects would be extremely useful. For instance, glucocorticoids have been largely used in vertebrates, through noninvasive sampling (e.g. faeces, feathers, saliva), as a general stress indicator to better understand the impact of environmental variables (Reeder and Kramer, 2005; Romano *et al.,* 2010; Fairhurst *et al.,* 2011). If such an analog does not seem to exist in insects, several elements of a general stress response have been studied in honeybees (e.g. biogenic amines, pathways of the energetic metabolism, heat shock proteins, vitellogenin) (Even *et al.,* 2012; Bordier *et al.,* 2017). Whether they could be transposed to other bee species remains to be investigated. However, this should be a reasonable target since moving physiological methodologies from model species (e.g. honeybees, bumblebees) to non-model species—and including physiological markers in monitoring programs—are becoming increasingly feasible (Vanderplanck *et al.,* 2021b).

In sum, the integration of physiological data into species distribution and abundance data has the potential to provide decidedly complementary points of view on how bees deal with environmental constraints and how their populations change. To achieve this goal, a clear reinforcement of multidisciplinary approaches (e.g. spatial and landscape ecology, physiology, taxonomy), combined with training, sharing of protocols and methods, as well as the development of standardized measures for physiological health of bees would be needed in future bee conservation studies. Therefore, in the short-term, assessing physiology might be more complex, expensive and time consuming than surveying species in the field. However, such an approach, especially with the identification of surrogate species, should lead to early indicators of bee population sensitivity to (positive or negative) environmental changes. Therefore, in the long-term, we hope the benefits will outweigh the costs, especially given that proactive conservation methods (i.e. acting before population size declines to critical levels) are considered more successful and cost effective than reactive strategies (Drechsler *et al.,* 2011).

## Data availability

No new data were generated or analysed in support of this article.

## Conflicts of interest

The authors declare that they have no conflicts of interest with this manuscript.

## Funding

This work was supported by the 2018–2019 BiodivERsA joint call for research proposals, under the BiodivERsA3 ERA-Net COFUND programme, and with the funding organization Agence Nationale de la Recherche in France [ANR-19-EBI3-0003].
